# Improved Optical and Morphological Properties of Vinyl-Substituted Hybrid Silica Materials Incorporating a Zn-Metalloporphyrin

**DOI:** 10.3390/ma11040565

**Published:** 2018-04-06

**Authors:** Zoltán Dudás, Eugenia Fagadar-Cosma, Adél Len, Loránd Románszki, László Almásy, Beatrice Vlad-Oros, Daniela Dascălu, Andraž Krajnc, Manfred Kriechbaum, Andrei Kuncser

**Affiliations:** 1 Wigner Research Centre for Physics, Institute for Solid State Physics and Optics, Hungarian Academy of Sciences, P.O. 49, 1525 Budapest, Hungary; len.adel@wigner.mta.hu (A.L.); almasy.laszlo@wigner.mta.hu (L.A.); 2 Institute of Chemistry Timisoara of Romanian Academy, Laboratory of Inorganic Chemistry, Bv. Mihai Viteazul, No. 24, RO-300223 Timișoara, Romania; efagadar@yahoo.com; 3 Faculty of Engineering and Information Technology, University of Pécs, Boszorkány Str 2, 7624 Pécs, Hungary; 4 Functional Interfaces Research Group, Institute of Materials and Environmental Chemistry, Research Centre for Natural Sciences, Hungarian Academy of Sciences, Magyar tudósok körútja 2., 1117 Budapest, Hungary; romanszki.lorand@ttk.mta.hu; 5 Faculty of Chemistry, Biology, Geography, Department of Biology-Chemistry, West University of Timisoara, 16 Pestalozzi, 300115 Timișoara, Romania; beatrice.vlad@e-uvt.ro (B.V.-O.); daniela.dascalu@e-uvt.ro (D.D.); 6 Department of Inorganic Chemistry and Technology, National Institute of Chemistry, Hajdrihova 19, 1001 Ljubljana, Slovenia; andraz.krajnc@ki.si; 7 Institute of Inorganic Chemistry, Graz University of Technology, Stremayrgasse 9, 8010 Graz, Austria; manfred.kriechbaum@tugraz.at; 8 National Institute of Materials Physics, 405a Atomistilor Street, 077125 Bucuresti-Magurele, Romania; andrei.kuncser@infim.ro; 9 Faculty of Physics, University of Bucharest, 405 Atomistilor Street, 077125 Bucuresti-Magurele, Romania

**Keywords:** vinyltriacetoxysilane, sol-gel technique, porphyrin, hybrid silica materials, physico-chemical characterization, optical properties

## Abstract

This work is focused on a novel class of hybrid materials exhibiting enhanced optical properties and high surface areas that combine the morphology offered by the vinyl substituted silica host, and the excellent absorption and emission properties of 5,10,15,20-tetrakis(*N*-methyl-4-pyridyl)porphyrin-Zn(II) tetrachloride as a water soluble guest molecule. In order to optimize the synthesis procedure and the performance of the immobilized porphyrin, silica precursor mixtures of different compositions were used. To achieve the requirements regarding the hydrophobicity and the porous structure of the gels for the successful incorporation of porphyrin, the content of vinyltriacetoxysilane was systematically changed and thoroughly investigated. Substitution of the silica gels with organic groups is a viable way to provide new properties to the support. An exhaustive characterization of the synthesized silica samples was realised by complementary physicochemical methods, such as infrared spectroscopy (FT-IR), absorption spectroscopy (UV-Vis) and photoluminescence, nuclear magnetic resonance spectroscopy (^29^Si-MAS-NMR) transmission and scanning electron microscopy (TEM and SEM), nitrogen absorption (BET), contact angle (CA), small angle X ray and neutron scattering (SAXS and SANS). All hybrids showed an increase in emission intensity in the wide region from 575 to 725 nm (Q bands) in comparison with bare porphyrin. By simply tuning the vinyltriacetoxysilane content, the hydrophilic/hydrophobic profile of the hybrid materials was changed, while maintaining a high surface area. Good control of hydrophobicity is important to enhance properties such as dispersion, stability behaviour, and resistance to water, in order to achieve highly dispersible systems in water for biomedical applications.

## 1. Introduction

Despite all the research over the last three decades in the field of sol-gel synthesis and applications [1,2,3,4,5,6,7] and technical and medical uses of porphyrins [8,9,10,11,12,13,14,15], the development of new materials exhibiting novel biological, optical and sensing properties [16,17,18,19] remains a great challenge. Combining the properties of silica as host with the special properties of the porphyrins has proved to be of great interest [20,21,22,23,24]. Obtaining porphyrin-silica hybrid nanomaterials that preserve or even enhance the optical and catalytic properties of the incorporated porphyrin is one of the main research targets [25,26,27,28]. In addition, advanced porphyrin-based hybrid nanomaterials characterized by high surface area, non-toxicity and biocompatibility, and providing functional groups capable of further derivatization, represent a novel approach to chemotherapeutic applications [29,30].

Substitution of the silica gels with organic groups is a viable way to provide new properties to the support [31,32,33] and also to induce a favourable effect upon the guest molecule’s properties and activity [34,35,36]. For example, vinyl derivatives are reported to offer the best activity towards cellulase immobilization in ordered mesoporous FDU-12 type silica with face-centered cubic structures [37,38]. Other vinyl substituted derivatives, such as vinyltrimethoxysilane (VTMS) and vinyltriethoxysilane (VTES) were also successfully used for the sol-gel entrapment of biomolecules [39,40,41,42].

Predominantly, VTMS and VTES are used to obtain vinyl substituted silica gels, therefore these materials are well-known and characterized [43,44,45,46,47,48]. We have chosen another vinyl substituted silica precursor, vinyltriacetoxysilane (VTAS), to expand the series of available hybrid materials. Up to now VTAS has been mainly used to obtain corrosion resistant coatings and silane bonded medical devices [49,50,51,52]. This precursor has been used only in a few cases for enzyme immobilization and this is the reason why we consider it has great potential and unexploited features [53,54].

Porphyrin based derivatives and hybrids possess optoelectronic properties that enable them to be used both in technical and medical applications, such as dye sensitized solar cells [55,56,57,58], sensors [59,60,61], catalysts [62,63] and photocatalysts [64,65,66], as well as photodynamic therapies for viruses and cancer [67,68,69].

The water solubility of porphyrin facilitates its incorporation in silica matrices, allowing the synthesis to remain as simple as possible without the need for additional organic solvent.

The goal of this study was to obtain a novel class of hybrid materials exhibiting enhanced optical properties by combining the morphology offered by the vinyl substituted silica host and the absorption and emission properties of 5,10,15,20-tetrakis(*N*-methyl-4-pyridyl)porphyrin-Zn(II) tetrachloride (Zn-TNMPyP) as a water soluble guest molecule.

**Figure 1:**
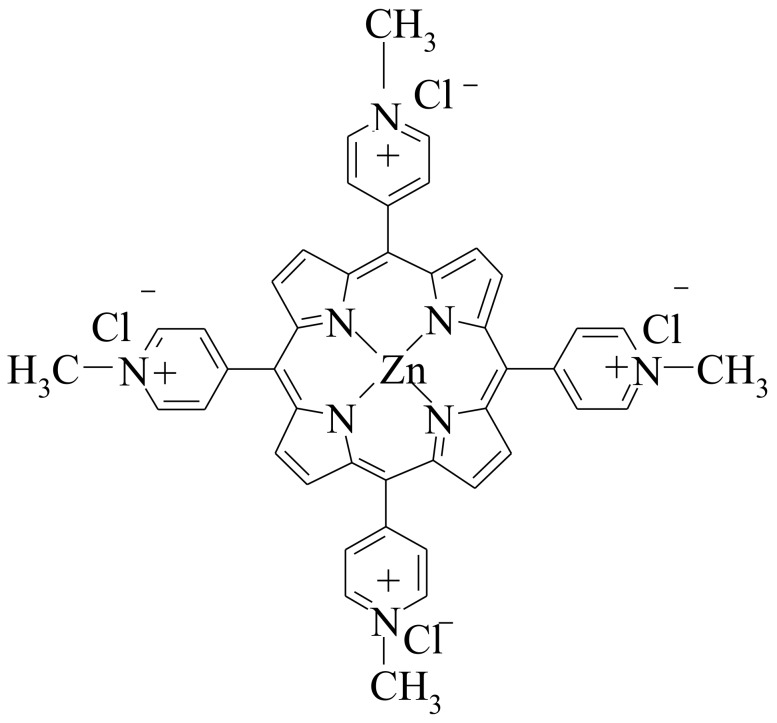
Water soluble (Zn-TNMPyP) metalloporphyrin used for incorporation [70]).

The selected porphyrin (Figure 1) was already proven to act as an optical pH sensor in the 5.5–10.5 domain with an accuracy of 0.03 pH units [70], which recommended it for providing enhanced optoelectrical properties in the novel hybrids.

In order to optimise the synthesis procedure and the performance of the immobilized porphyrin, silica precursor mixtures of different compositions were used. Varying the hydrophobicity of the gel, a suitable environment for successful porphyrin incorporation at minimal VTAS content could be achieved.

In order to achieve a comprehensive view of the structural properties of the synthesized silica samples complementary physicochemical characterisation methods were used. FT-IR and ^29^Si-MAS-NMR spectroscopy were used to determine the chemical composition, electron microscopy (TEM and SEM) to reveal the morphology, nitrogen adsorption, small angle X-ray and neutron scattering to gather information about the textural properties, contact angle for wettability measurements and UV-Vis and photoluminescence for the optical properties of the hybrid silica samples.

## 2. Materials and Methods

### 2.1. Materials and Methods

#### 2.1.1. Synthesis of Silica Xerogels

All reagents used in this work were of analytical grade and they were used as they were received from the supplier.

The novel hybrid silica xerogels have been synthesized starting from 0.03 mol of precursor mixture of VTAS/TEOS. The VTAS concentration was increased incrementally from 0 to 60 mol % (0, 5, 10, 20, 30, 40, 50 and 60). Silica sol was obtained by adding 0.15 mol of each precursor mixture in water and ethanol (molar ratio water/ethanol = 6/10). The hydrolysis of the silica sol was induced by HCl as acid catalyst. After one hour of magnetic stirring, the gelation was initiated by 1 M solution of NaF (A series) or NH_4_F (B series). This moment was considered as the starting time for the gelation. The HCl/F^−^ molar ratio was set to 1/8000. The obtained wet gels were left to age for 24 h, then dried for 10 h at 40, 60 and 105 °C, successively. The content of the precursors and the basic catalyst type used during the synthesis of xerogels for the A and B series of samples are listed in Table 1.

#### 2.1.2. Porphyrin Derivative

5,10,15,20-Tetrakis(*N*-methyl-4-pyridyl)porphyrin-Zn(II)tetrachloride, a water soluble tetrapyrrolemacrocycle, was synthesized by refluxing the symmetrical porphyrin base, meso-tetra(pyridyl)porphyrin, with an excess of methyl tosylate in *N*,*N*-dimethylformamide (DMF) for 8–10 h [71,72,73]. In order to improve the water solubility of the product, the tosylate counter-ion was ion-exchanged by chloride ion. The metalation was performed using a large excess of Zn salt in water/DMF to finally obtain Zn-TNMPyP [74,75].

#### 2.1.3. Immobilization of Zn-TNMPyP in Silica Xerogels

Due to the higher specific surface areas the B series gels were selected for Zn-TNMPyP immobilization. In each experiment, 3 mL of 10^−4^ M ZnTNMPyP aqueous solution was added to the hybrid silica sol before the initiation of the gelation, setting the final concentration of the porphyrin dye in the hybrids to ~10^−5^ M. 

### 2.2. Characterization of Samples

The morphology of the hybrid silica xerogels was observed with high-resolution transmission electron microscopy (JEM-ARM 100F) and scanning electron microscopy (Quanta FEG 250FEI, USEDAX analyzer detector BSE (back scattered electrons) low vacuum mode). The textural properties, porosity, specific surface area, and pore volume were measured by nitrogen adsorption-desorption at 77 K (Quantachrome Nova 1200e) and by small angle neutron (Yellow Submarine, Budapest Research Reactor, detailed in the Supplementary Materials (chapter S1.)) and X-ray scattering (high-flux SAXSess camera by Anton Paar, Graz Austria, for details see Supplementary Materials (chapter S1.)). Information on the chemical structure was collected from FT-IR spectra (JASCO 430 FT-IR) and UV–Vis spectra of the solid samples, recorded in the 300–800 nm range on an UV–Vis JASCO V-650 apparatus. Emission spectra (LS-55, Perkin-Elmer/UK) were recorded in the wavelength range 500–800 nm (100 nm/min, with an excitation maximum at 420 nm) at room temperature using a copper sample holder for solid samples.

For contact angle measurements, powder samples (both as-received and ground in a mortar) were deposited as a thin layer on double-sided adhesive tape stripes mounted on microscope slides placed on the stage of a home-built contact angle goniometer. Ultrapure water (Milli-Q, *ρ* = 18.2 MΩ∙cm) was used as the measuring liquid. Droplets of 2–10 µL were disposed from a 25 µL microsyringe (Hamilton) in multiple steps. Still images and videos were recorded during the disposal of the droplets. Left and right contact angles were measured. For each type of sample, 2–22 droplets were deposited, thus finally obtaining 2–22 values of contact angles. 

## 3. Results and Discussions

### 3.1. Gelation Time

**Figure 2:**
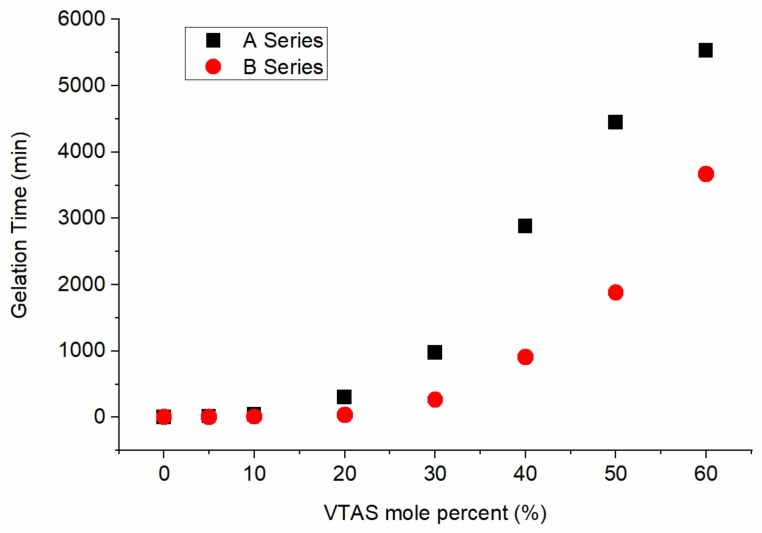
Evolution of the gelation time when using different molar ratios of precursors and NaF (A Series) or NH_4_F (B Series) catalysts.

As expected, the gelation time of the silica sols increased exponentially with the increasing molar percent of the VTAS. Faster gelation was obtained when using the NH_4_F catalyst (Figure 2). 

It is well-known that the condensation rate of silica precursors is favoured in comparison with their hydrolysis rate as the basicity of the medium is increased [76], and F^−^ ions significantly improve the hydrolysis and condensation, acting as a catalyst for both processes [77].

### 3.2. Textural Properties

#### 3.2.1. Nitrogen Adsorption

In case of the xerogels obtained from solely TEOS and from TEOS with addition of low amounts of VTAS (5% and 10%) IVb type isotherm were registered (Table 2). The addition of the VTAS changed the hysteresis type from H3/H1 to H4 (Figure S1, see details in Supplementary Materials). The addition of VTAS of higher than 20% causes the isotherm type to change to type Ib, which is typical for materials with wider micropores and narrow mesopores (<~2.5 nm), according to the last recommendation of IUPAC [78].

**Figure 3:**
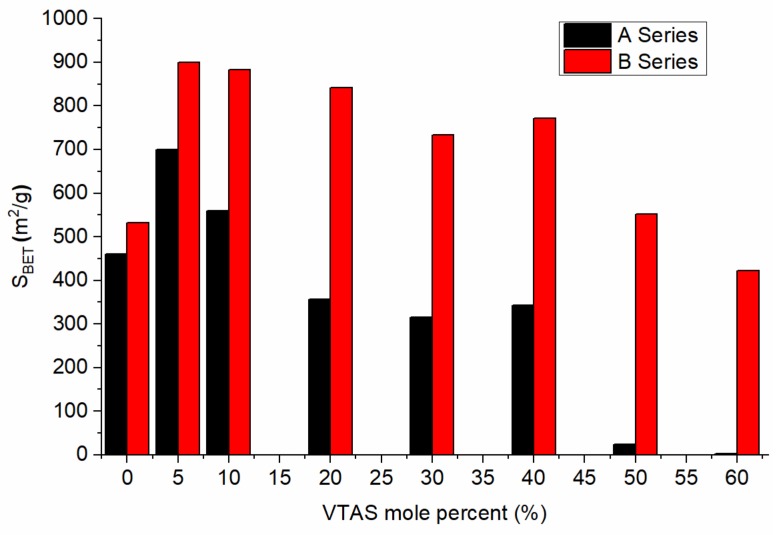
Influence of the molar ratios of the precursors and the types of catalysts upon the specific surface area of the synthesized silica gels.

All the obtained isotherms were characteristic for the micro-mesoporous and mesoporous materials. The surface areas (S_BET_) for the A and B series as a function of composition are presented in Figure 3. Similar behaviour depending on the composition was previously observed [79], however, in our case, much higher values of the specific surface areas were obtained using NH_4_F catalyst (B series). This is in agreement with the research data that indicates that F^−^ is solely responsible for the changes in morphology. The decrease in the pore size (Dp) is conditioned by a lower F^−^ ion concentration, that slows down the hydrolysis processes and triggers condensation As a consequence, smaller silica oligomers are produced having access into the already formed larger silica cores.

As expected, for the majority of the samples, the porphyrin immobilization in vinyl substituted silica gels caused a reduction of the specific surface area (Table 2) most probably due to the incorporation of the porphyrin into the pores of silica materials, in accordance with our previous studies [80]. Two exceptions could be identified: B60-VTAS, where the specific surface remained almost the same, and the unsubstituted silica gel (B00-VTAS), which showed a higher specific surface area after immobilization. By using 20, 40, and 60% of VTAS in precursor mixtures the microporous character became dominant, while 52–65% of the total pore volume (Vp) is made of micropores. A general observation is that the specific surface, the total volume of the pores and the volume of micropores have increased values when NH_4_F is used as a catalyst and the highest value is obtained when using 20% of VTAS. Similar tendencies regarding the evolution of the micro pore volume for the series of samples without and containing porhyrin were noticed. The only difference was that the micro pore volume was lower in the case of porphyrin incorporation (as can be seen in Figure S3 from Supplementary Materials).

All the samples containing porphyrin have a high specific surface area that recommend their further use as sensing or adsorbing materials.

#### 3.2.2. Small Angle Scattering

**Figure 4:**
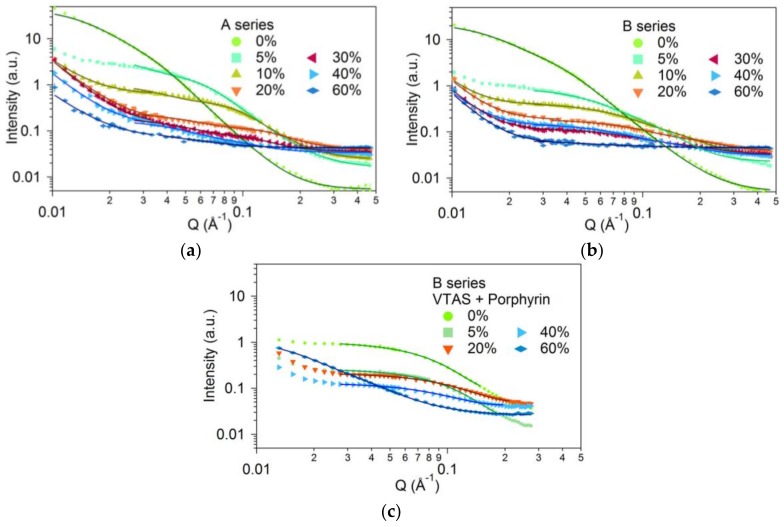
SANS curves of the measured A and B series of samples without (**a,b**) and with porphyrin (**c**). The percentages on the graphs refer to the molar ratio of VTAS.

SANS and SAXS scattering curves of the xerogels showed similar overall behaviour (Figure 4 and Figure S4 from Supplementary Material). Using simple phenomenological models, the structure was described as a network of aggregated particles with rough surfaces.

For the primary particles formed in the sol-gel process, the scattered intensity was related to their size, or radius of gyration, R_g_ according to (1):

(1) $$I\left(Q\right)=A\;exp\frac{-Q^{2}{R_{g}}^{2}}{3}$$

where A is proportional to the volume fraction of the scattering objects, and to the square of the contrast. R_g_ is a characteristic size of the particle, defined in analogy to the mechanical gyration radius. This approximation is generally valid for the small *Q* range with QR < 1 (Guinier behaviour), where *Q* is the magnitude of the scattering vector. For larger *Q*, scattering curves describe the surface characteristics of the scattering objects, according to (2):

(2) $$I\left(Q\right)=B{\left(\frac{1}{Q}\right)}^{p}$$

The exponent *p* in (2) describes the surface of the particles, in terms of a fractal model. For surface fractals 3 < *p* < 4 the fractal dimension is: D_S_ = 6 − *p*. Coefficients A and B are related to the number density and scattering length density of the particles and can be treated as adjustable scaling parameters. The SANS curves were fitted by the combination of these models, known as the unified model that was introduced by Beaucage [81]. It should be noted that, on the one hand, such interpretation is not unique as both the particles and pores can be regarded as scattering objects, on the other hand, their size is correlated since the holes are the cavities between the particles. Due to the possible comparison with the porosity, obtained by nitrogen adsorption, the measured dimensions are regarded as pore size.

In Figure 4 the SANS curves are shown for the three different series of samples. Figure 4a,b present the scattering intensities for the A and B series of samples, both without the addition of porphyrin, while in Figure 4c the curves for the B series with the addition of porphyrin are presented.

The A and B series of bare silica hybrids showed similar behaviour. The p exponents, describing the character of the surface of the pores and grains were obtained between 3.07 and 3.58 for the A series, and between 3.69 and 3.96 for the B series. Both series show characteristically fractal surfaces; the larger exponents (closer to 4) correspond to smoother pore surfaces. The porphyrin containing samples within the *Q* range of the SANS measurement (0.01–0.3 Å^−1^) did not show evaluable fractal surface behaviour.

**Figure 5:**
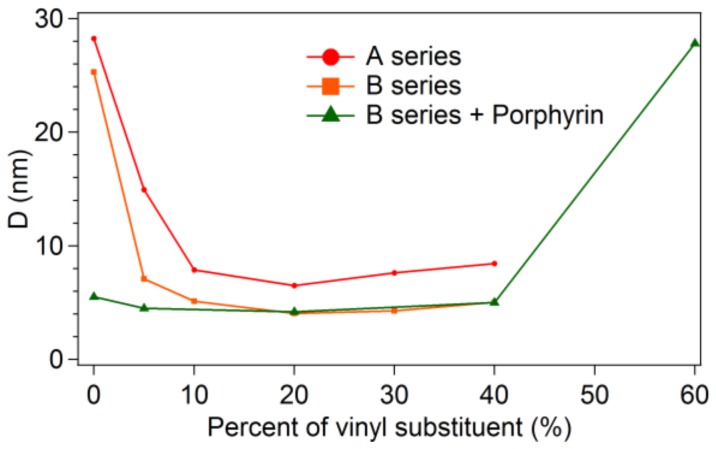
Average sizes obtained from the data fitting of the SANS curves versus the vinyl percentage.

For the same vinyl concentrations, the obtained sizes for the B series samples, with and without porphyrin, were smaller than those of the A series. This behaviour correlates to the shorter gelation time of the B series (Table 3). With the increasing vinyl content, the sizes of the pores decrease towards a minimum value for all three series at 20% vinyl substituent addition (Figure 5).

For higher vinyl content, the character of the scattering curves changes and the particle size cannot be reliably extracted. Applying the same model, the apparent size increases nearly threefold, which can be attributed to the network organization of the primary particles, as seen from the shape of the scattering curves. The fitting results for these samples are only shown for the sake of completeness.

A marked difference between the vinyl-free and vinyl-containing samples is seen. The scattering intensity is about one order of magnitude higher for the A00-VTAS and B00-VTAS samples as compared to the remaining samples. The model fitting shows that by vinyl substitution the characteristic sizes decreased by two to three times; good agreement was obtained with the BET data (see Table 2).

It can be concluded that in the case of B series of samples, prepared with NH_4_F, smaller pores are obtained. The added porphyrin fills the pores but does not significantly change the pore size; this characteristic is always determined by the vinyl content of the samples, so that by increasing the quantity of vinyl the pore size is decreased.

### 3.3. Morphologic Characterization

#### 3.3.1. TEM

**Figure 6:**
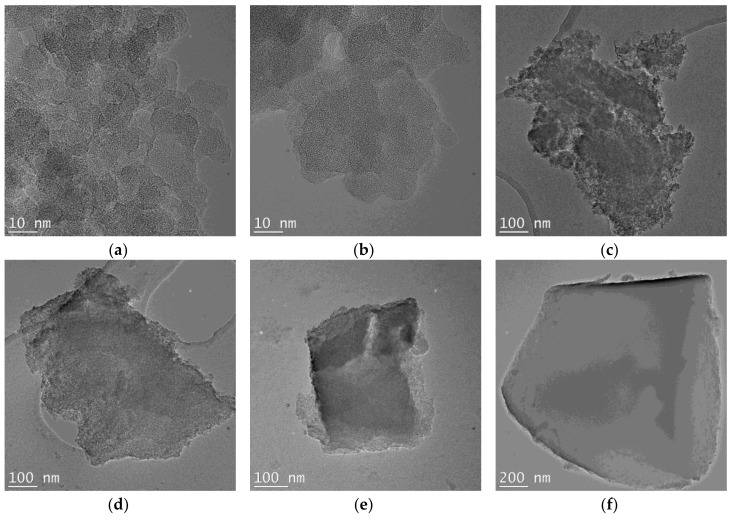
TEM micrographs of the hybrid silica-gels using different amount of vinyl substituents ((**a**) B00-VTAS, (**b**) B05-VTAS, (**c**) B05-VTAS, (**d**) B20-VTAS, (**e**) B40-VTAS, (**f**) B60-VTAS).

Significant variation of the VTAS concentration determined important morphologic changes of silica hybrids, as shown in TEM images (Figure 6). High magnification images of B00-VTAS and B05-VTAS showed similar porous morphology, with aggregated amorphous nanoparticles with sizes around 10 nm; by increasing the VTAS concentration the porosity decreased and the morphology became smoother (B20-VTAS and B40-VTAS). The high concentration VTAS substituted (B60-VTAS) sample showed the most compact and smooth morphology, i.e., the lowest porosity.

For the samples synthetized using only TEOS, for both series, almost the same value of the characteristic particle dimension (10–15 nm) was determined (Figure S5 detailed in Supplementary Materials).

#### 3.3.2. SEM

**Figure 7:**
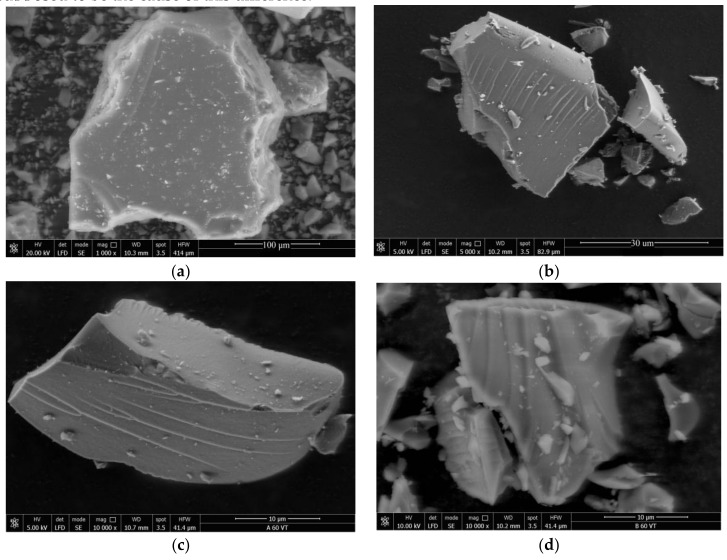
Evolution of the morphology of the hybrid silica gels distinguished by SEM images for samples from A and B series ((**a**) B05-VTAS; (**b**) B20-VTAS; (**c**) A60-VTAS; (**d**) B60-VTAS).

The morphologies of the xerogels obtained with precursors of different molar ratios were observed via SEM imaging (Figure 7). Comparing the A and B series from the samples of 60-VTAS, a deeper wave-like pattern was observed for the B series. The shorter gelation time for the B series is supposed to be the cause of this difference.

### 3.4. Chemical Composition

#### 3.4.1. FT-IR Spectroscopy

The IR spectra and the band assignments are presented in Supplementary Materials (Figure S6 and Table S1 from Supplementary Material). They contained all the characteristic vibration bands of silica gels, namely, bands corresponding to pure silica, vinyl and –OH groups. The large band between 3000 cm^−1^ and 3600 cm^−1^ is due to the overlapping of different –OH group vibrations and decreased with the increasing quantity of the vinyl substitution. This indicates an increasing hydrophobicity of the silica gels, in line with the contact angle results.

The materials exhibited similar shapes and location of the bands, with peaks around 1070, 595 and 460 cm^−1^, assigned to asymmetrical and symmetrical stretching, bending and deformation vibrations of Si–O–Si from silica. The 1070 cm^−1^ centered band moved to lower values with the increasing quantity of the vinyl substitution; such a shift is usually attributed to longer Si–O–Si distances [82].

Around 800 cm^−1^, a specific band for the silanol groups bending vibration could be observed; this band is present only for the bare silica sample and the samples with a low quantity of vinyl substitution.

The introduction of only a small amount of VTAS caused the appearance of the bands situated at 3068, 2985, 1411 cm^−1^, which is characteristic of the asymmetric and symmetric stretching and scissoring vibrations of the CH_2_.These bands were accompanied by a band at 1604 cm^−1^ caused by the stretching of the C=C bond. As expected, with the increasing amount of VTAS these bands’ intensities increased as well.

#### 3.4.2. Solid-State NMR

**Figure 8:**
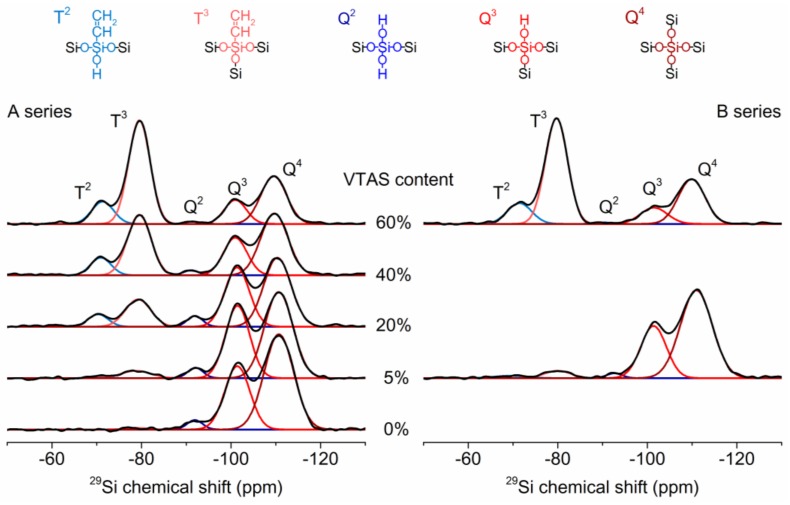
^29^Si MAS NMR spectra of vinyl substituted silica xerogels.

The ^29^Si MAS NMR spectra (Figure 8) showed an evolution of the peaks according to the molar ratio of the silica precursors. All of the xerogels were fully hydrolysed, as no evidence was found for the Si–CO_2_CH_3_ or Si–OC_2_H_5_ groups. The highest complete condensation (Q^4^ + T^3^) was obtained in the cases of the silica gels with maximum vinyl substitution (A60-VTAS and B60-VTAS) (Table 4). The introduction of a small amount of vinyl appeared to cause disordering in the silica network formation, with the lowest condensation level of ~58% (B05-VTAS and B20-VTAS).

**Figure 9:**
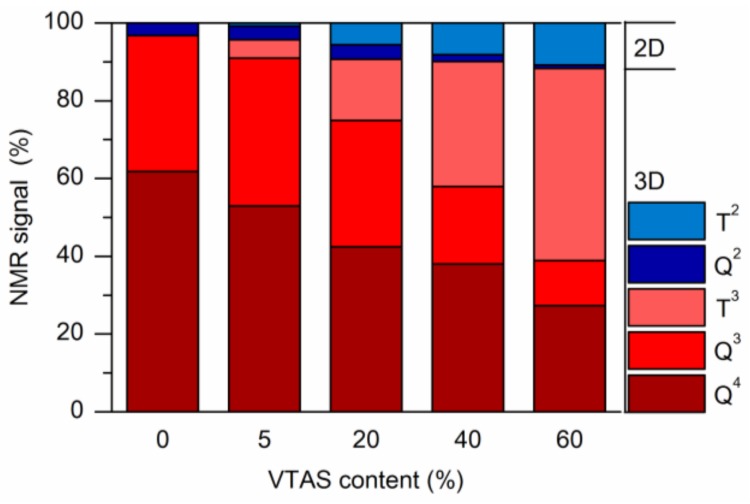
3D and 2D evolution of the silica network with the vinyl content.

In the case of samples synthesized with 0% and 5% VTAS, high amounts of Si atoms participated in the three-dimensional cross-linking gel network (Q^4^ + Q^3^ + T^3^ = 95–97%), while for the B20, B40, B60 or A60 samples the three-dimensional cross-linking had a constant value of ~90%. The linear silica network is indicated by the sum of the Q^2^ and T^2^ signals (Figure 9). The proportion of the linear silica network increased gradually from 3% to 12% as the amount of substituent increased.

The sum of the silica sites with –OH groups (Q^3^, Q^2^, T^2^) obtained for samples A05-VTAS and B05-VTAS are 33.1% and 42.3%, respectively. The 9% higher value of the –OH groups in the B series explains the lower hydrophobicity of this sample. For the same reason 10-VTAS, 20-VTAS and 30-VTAS samples in the B series have lower hydrophobicity, as also supported by the FT-IR spectra (Figure S6 from Supplementary Materials) and confirmed by the contact angle results.

##### NMR Correlation Experiments

**Figure 10:**
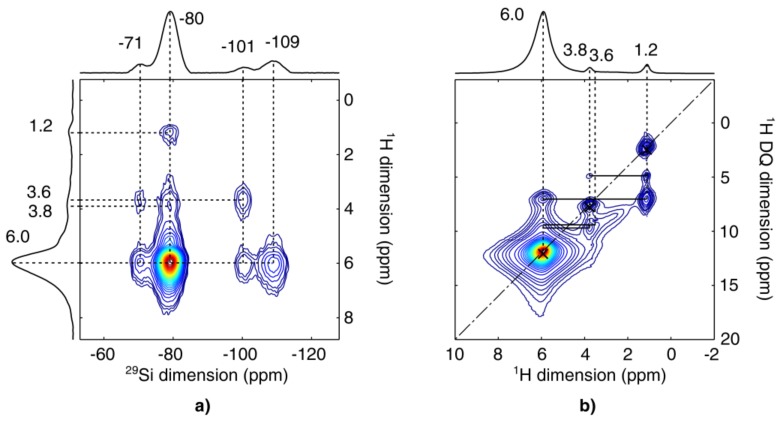
(**a**) ^1^H–^29^Si HETCOR and (**b**) ^1^H DQ-SQ BABA MAS NMR spectra of B60-VTAS sample.

2D ^1^H–^29^Si heteronuclear correlation (HETCOR) NMR measurements were carried out to characterize the proton environment of the silicate chains (Figure 10a). The ^1^H peaks located at 1.2 ppm and 3.8 ppm can be attributed to methyl and ethyl groups of ethanol, respectively. More importantly, there is another peak located at about 3.6 ppm, which can be assigned to the –OH groups [83]. Strong correlations between protons at 3.6 ppm and Si nuclei at −71 ppm and −101 ppm suggest that –OH groups are attached to T^2^ and Q^3^ sites, and that there are no Si–OC_2_H_5_ groups present. It seems that ethanol is adsorbed to the silica network since cross peaks at 1.2 ppm for all the silicon sites were observed; with the one at −80 ppm being most easily noticed due to the highest content of T^3^ in the sample. The most intensive ^1^H peak, at 6 ppm, is assigned to the protons in vinyl groups. As expected, these protons are in close proximity with the attached T sites (cross peaks at −71 ppm and −80 ppm), as well as the Si atoms of the neighbouring Q sites (−101 ppm and −109 ppm).

The 2D ^1^H DQ-SQ homonuclear correlation spectrum obtained for B60-VTAS is illustrated in Figure 10b. The spectrum exhibits intense on-diagonal cross peaks which confirm the correlations between equivalent protons of the multi-proton groups, in our case CH_3_ (at 1.2 ppm), CH_2_ (at 3.8 ppm) and vinyl groups (6 ppm). The most important contribution comes from the interaction between protons of vinyl groups and –OH groups, which is further confirmation that –OH groups are attached to T^2^ Si nuclei. As expected, there is no diagonal peak at 3.6 ppm since the -OH groups are isolated and their protons are not coupled together. In addition, correlations between protons of vinyl groups and protons of the adsorbed ethanol were noticed.

**Figure 11:**
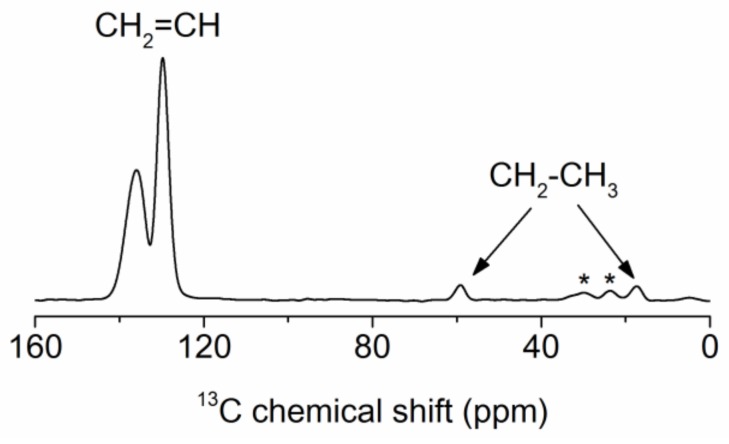
^1^H–^13^CPMAS spectra for sample B60-VTAS. Asterisks denote spinning sidebands.

^1^H–^13^C cross-polarization (CP) MAS NMR confirms that beside vinyl carbons (**C**H at 130 ppm; **C**H_2_ at 136 ppm) there is a very small amount of ethanol (**C**H_2_ at 59 ppm; **C**H_3_ at 17 ppm) present in the samples (Figure 11).

### 3.5. Contact Angle (CA) Measurements

**Figure 12:**
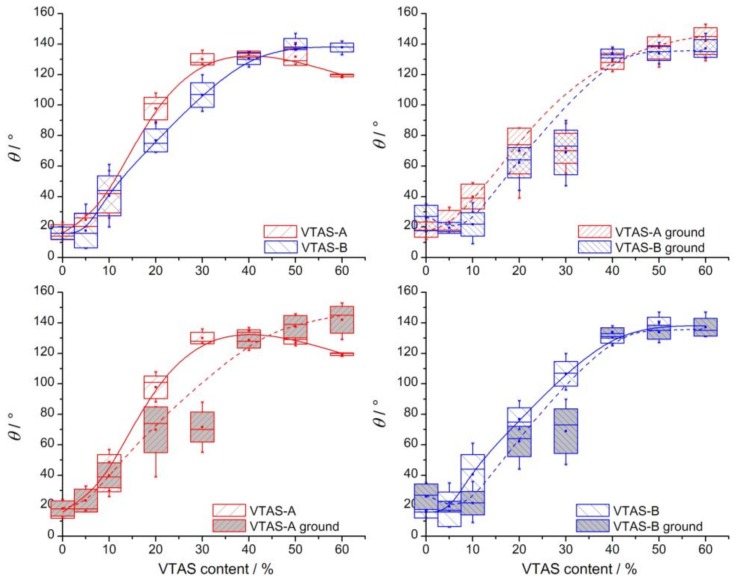
Box plot statistical representation of the advancing contact angles of VTAS samples, type A and B, both as-received and ground, in function of the vinyl content, grouped in pairs in order to facilitate comparison.

The surface wetting properties of the two series of xerogels have been analysed by contact angle measurements. CA data for all types of samples are presented as box plots as a function of vinyl content (Figure 12). The data are presented by comparison of catalyst type (A vs. B) and the state of the powder (as-received vs. grounded).

Several factors have been identified that hamper an accurate determination of the CA: the liquid/solid contact line hidden by powder grains; no surface reflection due to surface roughness; vapour (air)/liquid interface is hidden by powder grains; the pinning of the contact line on powder grains (hydrophobic samples); rapidly decreasing drop height and CA due to suction induced by porosity (hydrophilic samples); and possible chemical heterogeneity of the powder grains themselves (e.g., inhomogeneous coverage of vinyl groups).

The obtained CAs may be biased by all the above factors. Nevertheless, the large number and the statistical processing of the data, the full range of available sample series between 0 and 60% vinyl content, the application of not only backlighting but illumination at different angles with different backgrounds, fast video recording, careful digital image enhancement and CA reading made it possible to draw meaningful conclusions from the obtained results. Median CAs range from ca. 20° (0% vinyl content, i.e., silica from pure TEOS) to ca. 140° (60% vinyl content). The high CA values imply superhydrophobic surfaces. However, a truly superhydrophobic surface must exhibit not only a high advancing CA, but also a high receding CA, thus very low hysteresis, and additionally, a small (0–5°) roll-off angle. Thus, for a thorough characterization, the receding angles are important. As pointed out previously [84,85], the characteristics of a surface often correlate better (i.e., more strongly) with the receding angles (de-wetting) than the advancing ones (wetting). Unfortunately, taking into account the powdery state of the samples, a sessile drop type measurement of neither the receding angle nor the roll-off angle was feasible.

In accordance with the FT-IR (Section 3.4.1) and NMR results (Section 3.4.2), the CAs generally increase with increasing of the vinyl content. In an oversimplified view, the surface of the silica particles can be regarded as a two (actually many) component system: a low CA pristine silica component, and a high CA vinyl group component. As the relative amount of the high CA component increases, the overall CA of the composite surface should also be increased in a fashion described by the Cassie–Baxter theory [86]:

(3) $$cos\theta =f_{1}cos\theta_{1}+f_{2}cos\theta_{2},$$

where θ denotes the resultant CA of the two-component composite surface with CAs $\theta_{1}$, respectively $\theta_{2}$ for the two components that cover $f_{1}$, respectively $f_{2}$ fraction of the total surface, so that $f_{1}+f_{2}=1$. According to this theory, the resultant CA should have an arccos dependence on the surface coverage of the component of interest (in our case, vinyl), let us say $f_{2}$. Instead of such an arccos shaped curve, all graphs show a sigmoid-like dependence of the CA on the vinyl content. The fact that the shape of the actually measured CA dependence is substantially different from this theoretical one indicates that the bulk vinyl content of the different samples is not identical to the surface vinyl coverage. 

The as-received VTAS-A series samples exhibit a CA decline at high vinyl contents (above 40%). This fact correlates well with the corresponding gelation time dependence on the VTAS content (Figure 2).

At identical vinyl content, higher CAs were measured for VTAS-A than for VTAS-B samples, both in as-received and grounded form indicating higher/denser vinyl, or, as confirmed by the FT-IR (Section 3.4.1) and NMR results (Section 3.4.2), lower/sparser –OH coverage in the A series than in the B series. This fact might be a consequence of the preparation of the samples and different behaviours in relationship to the catalyst, which in the B series favours a slightly higher percentage of hydroxyl groups, in this way diminishing the hydrophobicity. There is an inflection point in the contact angle data around 20% VTAS content that complements well the SANS results, indicating that the determined pore size is smallest around 20% VTAS content. Besides, BET analysis proved that the highest amount of microporosity is present at the same VTAS content (discussed for the B series).

At identical vinyl contents, the ground samples showed lower CAs than the unground ones, for both VTAS-A and VTAS-B. Upon grinding, the grain size of the powder, i.e., the roughness of the powder layer, was significantly reduced. CA depends on surface roughness. Although contested, the simplest, and still generally accepted theory of CA dependence on roughness is given by Wenzel [87]:

(4) $$\cos\theta_{W}=r_{W}\cos\theta_{Y},$$

where $\theta_{W}$ stands for the real (“Wenzel”) CA of the rough surface, $\theta_{Y}$ is the ideal equilibrium (“Young”) CA of the same flat surface, and $r_{W}$ is a dimensionless roughness factor, the ratio of the real surface area to its geometrical projection. According to this relation, for hydrophilic surfaces ($\theta_{Y}$ < 90°), a higher geometrical surface area leads to a lower real CA, whereas increasing the real surface area of hydrophobic surfaces ($\theta_{Y}$ > 90°) results in even higher CAs (a common concept when designing superhydrophobic surfaces).

### 3.6. Fluorescence and UV-Vis Spectra

**Figure 13:**
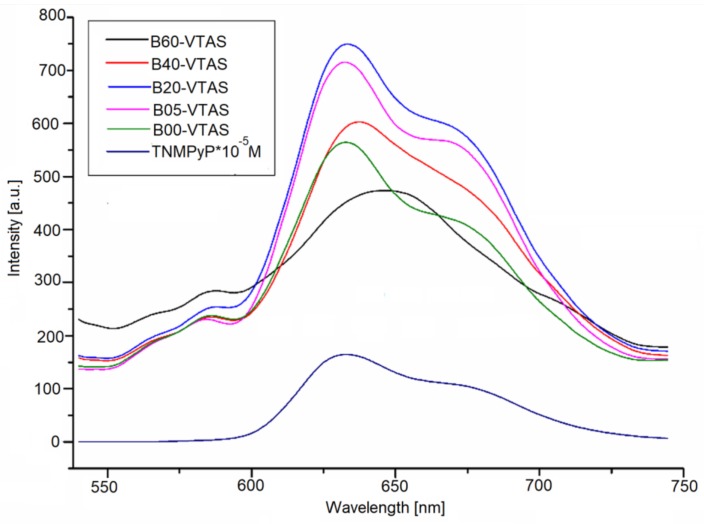
Emission spectra of the sol-gel immobilized Zn-TNMPyP using different vinyl substituted silica supports.

Photoluminescent emission spectra were recorded for all the samples containing porphyrin. In order to record the emission spectra (Figure 13), the samples were excited at 420 nm. The specific band of the porphyrin around 630 nm and an extended shoulder of lower intensity (around 670 nm), assigned to the porphyrin macrocycle, were observed. A comparison of the solely porphyrin emission spectrum with that of the hybrid materials revealed a significant increase in the intensity of the hybrids’ emission spectra, probably because the emission intensity is dependent on the distance and relative orientation of the molecular transition dipole moment with respect to the silica surface [88]. All the hybrids presented a shoulder on the most significant band in the proximity of 600 nm which is a characteristic of the Zn-metallated complex [89].

As can be seen in Figure 13, the emission intensity shows a Gaussian type evolution, with sample B20-VTAS presenting the highest intensity and sample B60-VTAS the lowest one. In the latter sample, the shape of the spectrum is entirely changed, with the two emission bands being merged into only one band, centred at 650 nm, which is typical for porphyrin Qx(0,0) transition.

**Figure 14:**
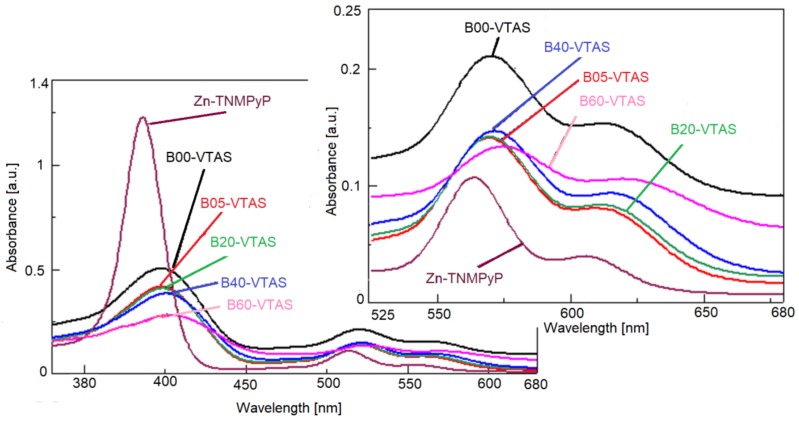
UV-Vis overlapped spectra of the sol-gel immobilized Zn-TNMPyP using different content of vinyl substituted silica supports. In detail, the Q bands of the samples.

Regarding the UV-Vis spectra of the Zn-porphyrin (Zn-TNMPyP) alone and immobilized by the sol-gel technique in silica matrices with different content of VTAS (Figure 14), several observations can be made. The spectrum of the porphyrin alone, registered in the water solution of concentration 10^−5^ M (the same concentration as in the hybrid samples), had the shape of metalloporphyrin with an intense and wide Soret band located at 440 nm and two Q bands of lower intensity, Q(0,1) at 565 nm and Q(0,0) at 607 nm, as expected. The Soret bands of all the porphyrin-silica hybrid materials were widened, bathochromically shifted, and decreased in intensity. Conversely, all the Q bands of the porphyrin-silica were red shifted and suffered a hyperchromic effect in comparison with the Zn-porphyrin. Comparing the UV-Vis spectra of the hybrid samples containing VTAS, the lowest intensity of absorption for both Soret and Q(0,1) bands is shown by B60-VTAS, the sample with the highest VTAS concentration.

The actual demand in PDT research is to obtain photosensitizers with an increased fluorescence quantum yield, which absorb at longer wavelength in the red region of the visible spectrum in order to reduce skin photosensitivity and to increase tumour selectivity [90]. On the other hand, regarding the absorption properties at 650 nm, the best hybrid material for PDT is B60-VTAS, which still has a better emission performance compared to the bare porphyrin.

## 4. Conclusions

The findings of this study consist in the achievement of hybrid materials with enhanced absorption and emission properties, having high surface area and tunable hydrophilic/hydrophobic balance, by combining the morphology offered by the vinyl substituted silica host and the benefits of (Zn-TNMPyP) porphyrin optical behaviour. 

Complex investigations by advanced structure-sensitive techniques, namely EM, SANS, SAXS, CA, NMR were performed in order to completely characterize the materials. 

All hybrids showed an increased intensity in emission in the wide region from 575 to 725 nm (Q bands) in comparison to bare porphyrin, which was attributed to the porphyrin immobilization into the vinyl substituted silica host. It was proven that the absorption properties showed the same trend with the intensity of absorption of the vinyl-substituted silica–porphyrin hybrids providing enhanced intensity of absorption in the same 530–680 nm region. Knowing that red absorption materials (λ > 630 nm) are considered the best second-generation photosensitizers for medical applications [91], these materials open up a wide range of applications in antibacterial and PDT studies.

A general observation is that the specific surface, the total volume of the pores and the volume of micropores have increased values when NH_4_F is used as a catalyst and the highest value is obtained when using 20% of VTAS.

Another finding of this study was that by simply tuning the VTAS content the hydrophilic/hydrophobic profile of the hybrid materials changed, while maintaining a high surface area.

By increasing the amounts of hydrophobic vinyl groups, the water contact angles of both A and B series of xerogels increased in a sigmoidal manner, with the contact angles of the A series being higher compared to the B series. The almost continuous increase in CA (and thus, of hydrophobicity) with VTAS content is in agreement with the observed gradual disappearance of hydrophilic IR-bands. The inflexion point, at about 20% VTAS content matches the extreme point locations of the NMR data and also the global minimum sizes as measured by SANS and SAXS. All samples that incorporated at least 40% VTAS can be considered hydrophobic materials.

## Figures and Tables

**Table 1:** Sol-gel synthesis parameters.

Silica Hybrid Sample (A Series) ^1^	Silica Hybrid Sample (B Series) ^2^	VTAS mol % in the Precursor Mixture (VTAS/TEOS)
A00-VTAS	B00-VTAS	0
A05-VTAS	B05-VTAS	5
A10-VTAS	B10-VTAS	10
A20-VTAS	B20-VTAS	20
A30-VTAS	B30-VTAS	30
A40-VTAS	B40-VTAS	40
A50-VTAS	B50-VTAS	50
A60-VTAS	B60-VTAS	60

^1^ The A seria used as catalyst the NaF and ^2^ the B seria used as catalyst NH_4_F.

**Table 2:** Comparison of the textural properties of the silica gels with or without Zn-TNMPyP.

Sample	Zn-TNMPyP	Isotherm and Hysteresys Type	Dp [ads] ^1^ (nm)	Dp [des] ^2^ (nm)	Dp [DFT] ^3^ (nm)	S_BET_ (m^2^/g)	Vp (cm^3^/g)	Vp_micro_ (cm^3^/g)
B00-VTAS	No	Iva–H3	3.64	13.35	7.31	531.3	0.147	0.025
	Yes	IVa–H1	3.64	4.35	5.69	695.6	0.756	0.000
B05-VTAS	No	IVa–H4	3.63	3.66	4.57	900.4	0.807	0.056
	Yes	IVa–H4	3.64	3.42	4.57	532.4	0.388	0.021
B20-VTAS	No	IVa–H4	3.63	3.65	2.58	842.0	0.488	0.305
	Yes	Iva–H4	3.63	3.09	4.57	657.0	0.373	0.202
B40-VTAS	No	Ib	3.66	3.62	2.54	771.6	0.453	0.276
	Yes	Ib	3.66	3.42	2.51	672.9	0.385	0.245
B60-VTAS	No	Ib	3.63	3.64	2.11	421.7	0.254	0.166
	Yes	Ib	3.63	3.43	2.11	428.2	0.278	0.145

^1^ Dp [ads]—Pore diameter from adsorption, ^2^ Dp [des]—Pore diameter from desorption, ^3^ Dp [DFT]—Pore diameter with DFT model.

**Table 3:** SANS and SAXS fitting parameters.

Vinyl Content	A Series		B Series		B Series + Porphyrin D (nm) SANS
	SANS	SAXS	SANS	SAXS	
	D (nm)	D (nm)	D (nm)	D (nm)	
0%	28.25 ± 0.46	20.15 ± 0.01	25.31 ± 0.29	16.15 ± 0.03	5.51 ± 0.02
5%	14.95 ± 0.05	7.97 ± 0.01	7.10 ± 0.06	7.10 ± 0.03	4.49 ± 0.01
10%	7.87 ± 0.04	5.37 ± 0.003	5.11 ± 0.04	5.17 ± 0.005	-
20%	6.50 ± 0.06	4.36 ± 0.02	4.04 ± 0.07	4.39 ± 0.01	4.20 ± 0.02
30%	7.64 ± 0.19	14.26 ± 0.5	4.28 ± 0.09	3.91 ± 0.01	-
40%	8.45 ± 0.36	13.85 ± 0.34	5.03 ± 0.11	4.57 ± 0.03	5.02 ± 0.04
50%	-		-	3.97 ± 0.2	-
60%	-		-	6.99 ± 0.21	27.30 ± 0.65

**Table 4:** The results obtained from the quantification of the ^29^Si CP-MAS NMR spectra.

Sample	Q^4^ (%)	Q^3^ (%)	Q^2^ (%)	T^3^ (%)	T^2^ (%)	Q^4^ + T^3^ (%)	Q^4^ + T^3^ + Q^3^ (%)	Q^2^ + T^2^ (%)	Q^3^ + T^3^ (%)
B00-VTAS	61.7	35.0	3.2	0.0	0.0	61.7	96.8	3.2	35.0
B05-VTAS	52.9	38.0	3.5	4.7	0.8	57.7	95.7	4.3	42.7
B20-VTAS	42.4	32.6	3.8	15.6	5.6	58.1	90.6	9.4	48.2
B40-VTAS	38.0	20.0	1.8	32.1	8.1	70.1	90.1	9.9	52.1
B60-VTAS	27.3	11.6	0.8	49.5	10.8	76.8	88.4	11.6	61.1
A05-VTAS	62.6	29.7	1.7	4.3	1.7	66.9	96.6	3.4	34.0
A60-VTAS	26.4	9.7	0.5	52.0	11.3	78.5	88.2	11.8	61.8

## Supplementary Materials

The following are available online at http://www.mdpi.com/1996-1944/11/4/565/s1, Figure S1. Nitrogen adsorption-desorption isotherms evolution in function of the vinyl substitution ratio; Figure S2. Pore size distribution from DFT method; Figure S3. Evolution of the total pore volume vs. micro pore volume for the samples without (a) and with porhyrin (b); Figure S4. SAXS curves of the A and B series of samples. Mole percentage of VTAS is shown in the legend. Symbols represent the measured data, lines are the fitted curves; Figure S5. Size distribution of nanoparticles determined from the TEM images; Figure S6. Comparative study of the FT-IR spectra obtained from using different VTAS/TEOS xerogels; Table S1. Assignment of FT-IR bands.
