# Supplementary material for: Improved Optical and Morphological Properties of Vinyl-Substituted Hybrid Silica Materials Incorporating a Zn-Metalloporphyrin
Source: Materials (Basel). 2018 Apr 6;11(4):565. doi: 10.3390/ma11040565 (PMC5951449; doi:10.3390/ma11040565)
Supplement: Supplementary file 1 [file materials-11-00565-s001.pdf]

Supplementary Materials: Improved optical and morphological properties of vinyl-substituted hybrid silica materials incorporating a Zn-metalloporphyrin

Zoltán Dudás, Eugenia Fagadar-Cosma, Adél Len, Loránd Románszki, LászlóAlmásy, Beatrice Vlad-Oros, Daniela Dascălu, Andraž Krajnc, Manfred Kriechbaum and Andrei Kuncser

S1. Measurements methods:

Nitrogen adsorption -Prior to the measurements, the samples were degased under vacuum for 6 h at 80 °C.

^{29}*Si MAS NMR* spectra were recorded using a π/2 excitation pulse with duration of 2.2 μs, 2400 repetitions and delay of 30 s. During the acquisition a high-power XiX heteronuclear decoupling was used on the ^{1}H channel. The sample spinning frequency was 10 kHz. ^{13}C CPMAS spectrum was acquired by first exciting protons with a π/2 pulse of 2.3 μs, followed by a 5.0 ms cross-polarization (CP) block and heteronuclear decoupling during the acquisition. The number of scans was 3800 with a delay of 1 s. The sample was spun at 16 kHz. Two-dimensional ^{1}H-^{29}Si HETCOR NMR spectrum was obtained at sample spinning frequency of 20 kHz. The duration of the CP block was 5.0 ms, the number of increments along the indirectly detected dimension was 64 and the number of scans for each increment was 1600 with a delay of 1 s. ^{1}H-^{1}H homonuclear correlations were measured using back-to-back recoupling sequence (BABA). Double-quantum coherence excitation and reconversion were realized using a single BABA cycle. The pulse width of 2.3 μs and delay of 1 s were used. The sample rotation rate and the spectral width of indirect dimension were 20 kHz and 40 kHz, respectively. 190 slices along indirect dimension with 48 transients each were accumulated. The Larmor frequencies for ^{1}H, ^{13}C and ^{29}Si were 599.55, 150.76 and 119.11 MHz, respectively. The frequency axes were referenced relative to the signal position of tetramethylsilane.

SANS

The mesoscale structure of the materials was measured with the SANS instrument Yellow Submarine at Budapest Neutron Centre. The covered Q range was: 0.01–0.5 Å^{-1}, the sample to detector distances were 1.3 m and 5.4 m, the wavelengths were 3.7 Å and 8.4 Å.

SAXS measurements.

Small angle X-ray scattering (SAXS) measurement was performed with a high-flux SAXSess camera (Anton Paar, Graz, Austria) connected to a Debyeflex 3003 X-ray generator (GE-Electric, Ahrensburg, Germany), operating at 40 kV and 50 mA with a sealed-tube Cu anode.

The Goebel-mirror focused and Kratky-slit collimated X-ray beam was line shaped (17 mm horizontal dimension at the sample) and scattered radiation from the samples (measured in the transmission mode) was recorded by an one-dimensional MYTHEN-1k microstrip solid-state detector (Dectris, Baden-Daettwil, Switzerland), within a Q-range (with Q being the magnitude of the scattering vector) of 0.01 to 0.5 Å^{-1}. Using Cu K_α radiation of wavelength 0.154 nm and a sample-to-detector distance of 309 mm, this corresponds to a total 2θ region of 0.14° to 7°, applying the conversion $Q = 4\pi(\sin\theta)/\lambda$ with 2θ being the scattering angle with respect to the incident beam and λ the wavelength of the X-rays. For our SAXS measurements the powdered samples were filled into a vacuum-tight foil-sealed flat sample-cell. The beam path through the sample was about 1 mm including the foils of 20 μm in thickness (polycarbonate). All measurements, also that of the empty cell, were done in vacuum and at ambient temperature (ca. 293 K) with an exposure time of typically 60–120 s. The scattering of the foils was subtracted from the scattering of the samples after normalizing the two patterns to the same transmission intensity.

S2. Results

S2.1. Nitrogen adsorption

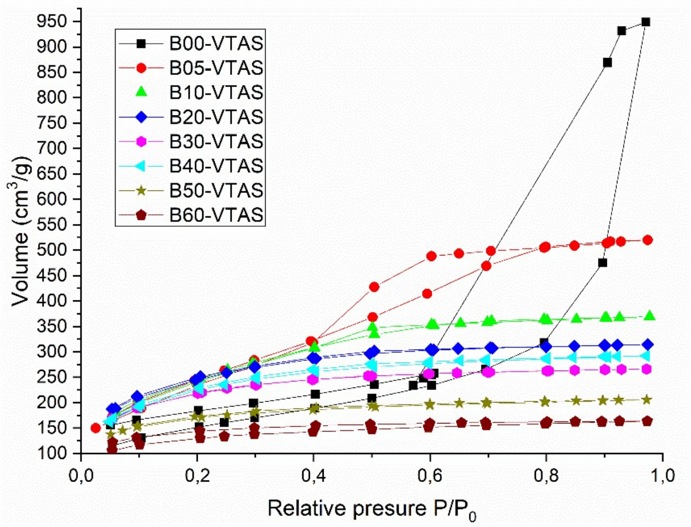

Figure S1. Nitrogen adsorption-desorption isotherms in function of the vinyl substitution ratio

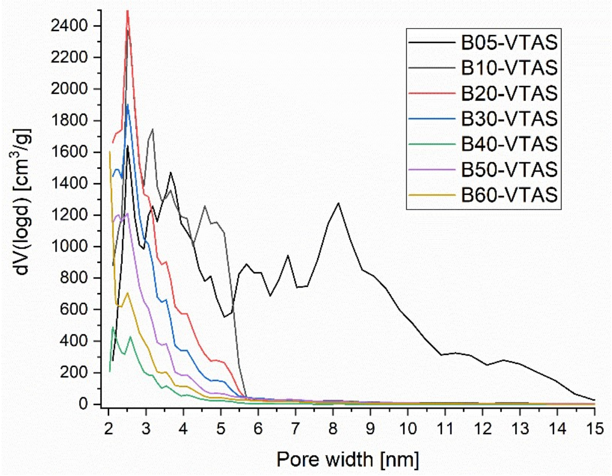

Figure S2. Pore size distribution obtained from DFT method

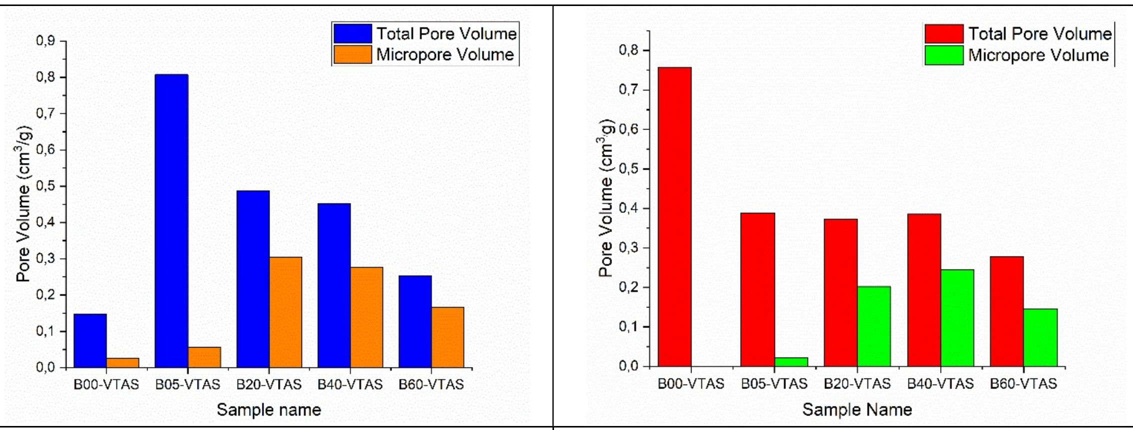

(a)	(b)

Figure S3. Evolution of the total pore volume vs micro pore volume for the samples without (**a**) and with porhyrin (**b**)

S2.2. SAXS

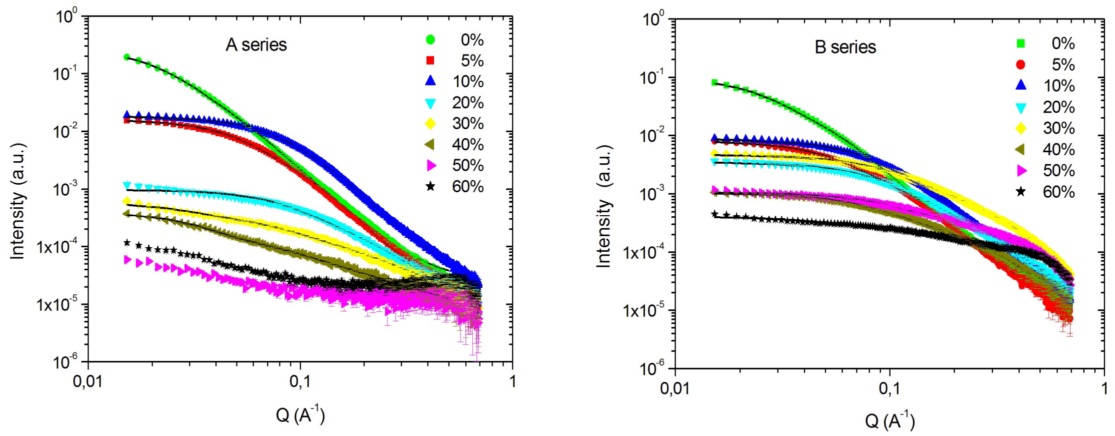

(a) (b)

Figure S4. SAXS curves of the A (a) and B series (b) of samples. Mole percentage of VTAS is shown in the legend. Symbols represent the measured data, lines are the fitted curves.

S.2.3. TEM

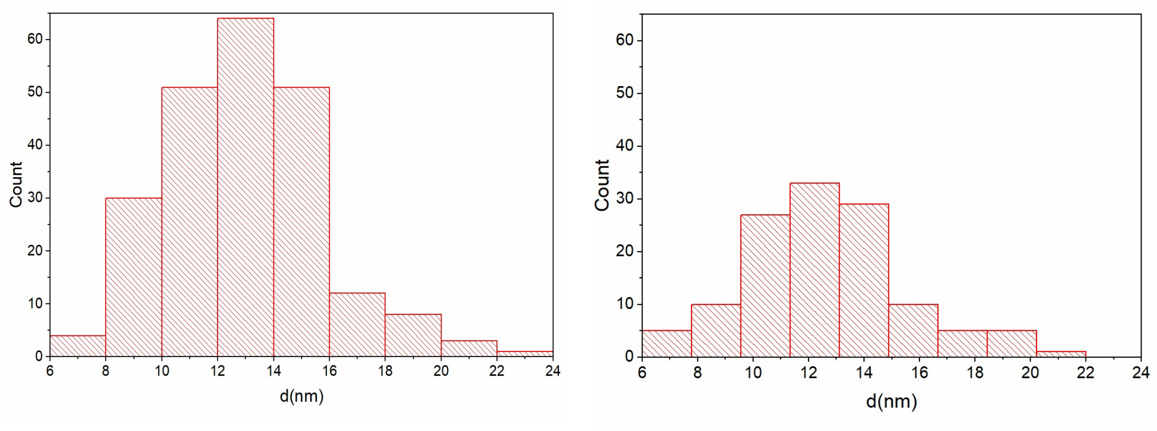

(a) (b)

Figure S5. Size distribution of nanoparticles determined from the TEM images. (**a**) A00-VTAS; (**b**) B00-VTAS.

S2.4. FT-IR Spectroscopy

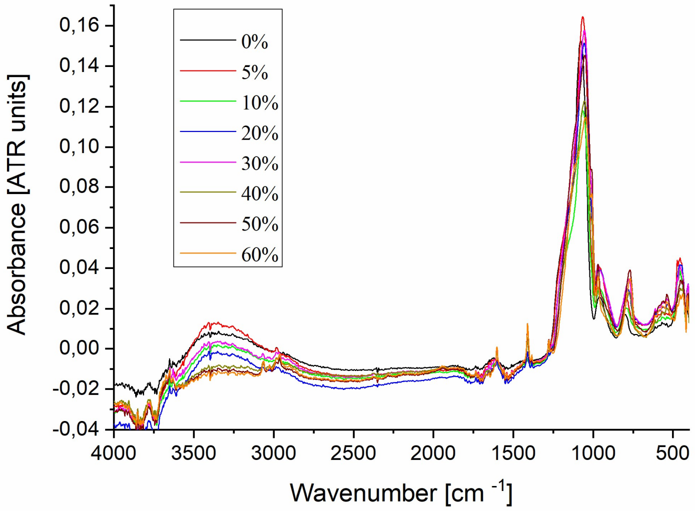

Figure S6. FT-IR spectra of the VTAS/TEOS xerogels with NH_4F catalysts (B series).

Table S1. Assignment of FT-IR bands.

Assignement	Band position
OH str.	3770-3780
O–H str. and adsorbed water	3650-3655
ν (OH), H-bond	3430
νa (CH_2), vinyl	3060-3065
Combination	3025-3028
νs (CH_2), vinyl	2987-3000
ν (CH), vinyl	2962-2964
ν (C=O)	1738
ν (C=C)	1600-1605
δ (CH_2), vinyl	1410
δ (CH), vinyl	1276-1278
νa (SiOSi)	1043-1045
τ (CH_2), vinyl	1010
ω (CH_2)	970
OH bending (silanol)	798-800
ν (SiC) (conformer A)	790-795
ν (SiC) (conformer B)	772-775
δ (OSiO)	595
δ (C=O)	572-576
δ (CH), vinyl	538-540
δs(CSiO)	450-455
